# Dechlorination of wastewater from shell-based glucosamine processing by mangrove wetland-derived fungi

**DOI:** 10.3389/fmicb.2023.1271286

**Published:** 2023-10-13

**Authors:** Zhiping Han, Edward S. X. Moh, André L. S. Santos, Iuri C. Barcellos, Yuanhuai Peng, Weicong Huang, Jianzhi Ye

**Affiliations:** ^1^College of Food Science and Engineering, Lingnan Normal University, Zhanjiang, Guangdong, China; ^2^ARC Centre of Excellence for Synthetic Biology, School of Natural Sciences, Macquarie University, Sydney, NSW, Australia; ^3^Department of General Microbiology, Institute of Microbiology Paulo de Góes, Federal University of Rio de Janeiro (UFRJ), and Rede Micologia RJ – FAPERJ, Rio de Janeiro, Brazil; ^4^Agricultural Products Processing Research Institute, Chinese Academy of Tropical Agricultural Sciences, Zhanjiang, Guangdong, China

**Keywords:** fungi, industry wastewater, inorganic chloride removal, bioremediation, environmental safety

## Abstract

Wastewater from processing crustacean shell features ultrahigh chloride content. Bioremediation of the wastewater is challenging due to the high chloride ion content, making it inhospitable for most microorganisms to survive and growth. In this study, mangrove wetland-derived fungi were first tested for their salt tolerance, and the highly tolerant isolates were cultured in shrimp processing wastewater and the chloride concentration was monitored. Notably, the filamentous fungal species *Aspergillus piperis* could remove over 70% of the chloride in the wastewater within 3 days, with the fastest biomass increase (2.01 times heavier) and chloride removal occurring between day one and two. The chloride ions were sequestered into the fungal cells. The genome of this fungal species contained Cl^−^ conversion enzymes, which may have contributed to the ion removal. The fungal strain was found to be of low virulence in larval models and could serve as a starting point for further considerations in bioremediation of shell processing wastewater, promoting the development of green technology in the shell processing industry.

## 1. Introduction

Inorganic chloride (Cl^−^) pollution is one of the problems faced by industries producing chitin, chitosan, and glucosamine from the cuticles of crustaceans such as shrimp, lobsters, and crabs, as the cuticles are only soluble in highly concentrated hydrochloric acid (Bastiaens et al., 2019). It has been reported that around 8.5 tons of 30% (v/v) hydrochloric acid has to be used for processing 1 ton of shrimp cuticles, resulting in a high concentration of Cl^−^ in the effluents (around 70 g/L; Bertuzzi et al., 2018). Currently, four strategies are primarily used in industry for removal of Cl^−^ from shrimp processing wastewater, including electrodialysis, precipitation, adsorption, and microporous separation (Association A.W.W, 2006; Sathasivan et al., 2017; Li et al., 2022). These strategies concentrate Cl^−^ as a result, and may cause salinization if improperly disposed (Li et al., 2022).

New technologies are emerging in two directions to prevent Cl^−^ pollution caused by shell processing. One is to develop a green method for chitin extraction, which has drawn lots of attention and can be basically divided into chemical and biological methods (Kozma et al., 2022). In the chemical approaches, alternative solvents, such as citric, acetic, and lactic acids as well as ammonium-based ionic liquids were used to extract chitin from shrimp shells (Cira et al., 2002; Tolesa et al., 2019; Kozma et al., 2022). Regarding biological approaches, enzymatic or microorganism fermentation methods have been employed to break down proteins and calcium carbonate in shells (Arbia et al., 2013; Hossin et al., 2021; Kou et al., 2021). The other direction is to develop alternative Cl^−^ removing methods from waters.

Bioremediation is environmentally friendly but not widely used in the Cl^−^ removal industries, since most bacteria commonly used in wastewater treatment are not resistant to >3 g/L Cl^−^ under normoxia (Liu et al., 2020; Li et al., 2022). On the other hand, biological removal of organic chlorine pollutants has been used and studied extensively with two common groups of prokaryotes, namely sulphate-reducing and denitrifying bacteria. Basically, these bacteria perform different adaptations to degrade chlorine pollutants, like aerobic fermentation on the surfaces of water or soil, denitrification response to changes in the oxygen concentration, and oxidation–reduction reactions in anoxic environments (Xu et al., 2019; Xing et al., 2020; Sobiecka, 2022). The marine bacterial species *Staphylococcus xylosus* has been reported to be able to convert Cl^−^ into organic cellular compounds and eventually lower the Cl^−^ concentration in wastewater (Abou-Elela et al., 2010). As an alternative to bacteria, one can also consider fungi as a potential candidate in Cl^−^ removal industries, as they can also be halophilic.

Microorganisms naturally growing in polluted environments are supposed to be capable of biodegrading toxic compounds (Sobiecka, 2022). Mangrove forest land may be an ideal location for isolating fungi capable in Cl^−^ removal from shrimp processing wastewater, though there has yet to be a report on this issue to our knowledge. Mangroves are a type of salt-tolerant submerged sclerophyllous plant species growing in coastlines worldwide at low tide areas in the tropics and subtropics. Mangrove forests form an interface between terrestrial and marine habitats and provide excellent support for migrating waterbirds, offshore fish, and other water fauna (Thatoi et al., 2013). The wetlands in mangrove forests feature not only high salt concentration (0.5–80 g/L) but also abundant macronutrients from decomposition, providing a unique biosphere for colonization of halophilic microorganisms (Schmitz et al., 2009; Hamzah et al., 2018; Jia et al., 2020).

Till now, plenty of filamentous fungi have been isolated from mangrove forest wetlands, including several species belonging to the following genera: *Penicillium, Aspergillus, Trichoderma, Stemphylium, Talaromyces, Setophoma, Mucor, Lasiodiplodia*, *Annulohypoxylon*, *Rhytidhysteron*, *Phomopsis*, *Diaporthe*, *Neosartorya*, *Beauveria*, *Eupenicillium*, and *Dipodascus* (Bonugli-Santos et al., 2015; Ancheeva et al., 2018; Jia et al., 2020). Similarly, yeast have been recovered from this habitat, particularly species belonging to the *Candida, Kluyveromyces, Pichia*, *Kodamaea*, *Debaryomyces*, and *Williopsis* genera (Chi et al., 2012; Bonugli-Santos et al., 2015). Among these fungi, some of them have been studied on their industrial application. For example, *Aspergillus sclerotiorum* (strain CBMAI 849) and *Mucor racemosus* (strain CBMAI 847) are able to degrade polycyclic aromatic hydrocarbons (Passarini et al., 2011; Bonugli-Santos et al., 2015). *Aureobasidium* sp. (strain P6), *Penicillium janthinellum* (strain P1) and *Tinctoporellus* sp. (strain CBMAI 1061) are able to decolor various synthetic dyes such as bromothymol blue, eriochrome black T, crystal violet, malachite green, and methyl orange (Chen et al., 2014; Rodriguez et al., 2015; Lu et al., 2018; Aung et al., 2019).

In this study, fungi growing near a sewage drain outlet in mangrove wetlands located in an industrial park in Zhanjiang, the southernmost city on the coast of mainland China, were isolated and studied in terms of fungal species, salt tolerance, chloride removal capacity and virulence. The results have the potential to offer valuable insights into the application of fungal strains in the remediation of Cl^−^ in the processing industry.

## 2. Materials and methods

### 2.1. Sampling campaigns

Sampling was conducted on three occasions in total over five afternoons before the tide rising, at three sites (seaward zone, mid zone and landward zone) in the mangrove forest (Leizhou, China, 21°68’N, 110°33′E), where soil samples were collected from 0 to 10 cm below the surface sediments. The samples were collected using a sterilized hand shovel (around 4 cm × 6 cm, with 18 cm handle) and large debris was removed by hand. All the samples from all sites were combined and mixed thoroughly before transferring into sterile 500 mL canning jars and transported to laboratory in an icebox.

### 2.2. Fungal isolation assay

Fungal isolation was carried out immediately after arrival, around 30 min after the collection, following Ahumada-Rudolph’s method with some modifications (Ahumada-Rudolph et al., 2016). Briefly, the sediment was suspended in 4 L sterile seawater, homogenized, and left to settle. The supernatant was collected by centrifugation at 100 g for 15 min and stirred well afterwards. Supernatant (2 mL) was diluted in series with sterilized seawater (10–100 folds in 10-fold intervals) with two replicates. An aliquot of 100 μL was transferred to yeast extract peptone dextrose agar (YPD) prepared using sterilized seawater supplemented with ampicillin, kanamycin and streptomycin (final concentration of 50 μg/mL, 50 μg/mL and 25 μg/mL respectively). The aliquot was spread onto the agar using a sterile glass coating rod and the incubation was conducted at 28°C.

Subcultures were performed till individual colonies were obtained. Morphology of individual colonies was verified under the microscope. Spores of isolated fungi were collected using sterilized water with 0.05% Tween 80 and stored at 4°C for up to 1 week. The agar with fungal growth was sliced off from the dish using sterilized scalpel (about 4 mm × 4 mm each piece), submerged in 80% glycerol solution (v/v) and stored at −80°C for 4 months.

### 2.3. Molecular methods for fungal identification

Fungal genomic DNA was extracted using the Qiagen DNEasy Plant Extraction kit (Qiagen Inc., Valencia, CA, United States). Primers (ITS1: 5’-TCCGTAGGTGAACCTGCGG-3′ and ITS4: 5’-TCCTCCGCTTATTGATATGC-3′) were used to amplify by polymerase chain reaction (PCR) the whole region of ribosomal internal transcribed spacer (*ITS1-5.8 s-ITS2*; Korabecna, 2007). The PCR reaction mixture included 10 μL of PCR master mix (Promega, 2X), 1 μL of the forward primer ITS1, 1 μL of the reverse primer ITS4, 1 μL of DNA extraction, and 7 μL of ddH_2_O. The thermal cycler was programmed with the following conditions: initial denaturation at 94°C for 4 min, followed by 35 cycles of denaturation at 94°C for 20 s, annealing at 55°C for 20 s, and extension at 72°C for 1 min, concluding with a final extension at 72°C for 10 min. PCR products were purified using the QIA quick PCR purification kit and sent to Sangon Biotech (Shanghai, China) for bidirectional sequencing. The assembled sequences were analyzed using BLAST^1^ against the standard database on NCBI.

### 2.4. Salt tolerance assay

Salt tolerance assay was performed on YPD agar supplemented with sodium chloride (NaCl, w/v) at different concentrations (50, 100, 150, 200 and 250 g/L). The spores of isolated fungi were inoculated on the salt-contained agar, cultured at 28°C for up to 10 days, and the fungal growth was monitored.

### 2.5. Cultivation of salt tolerance strains in the wastewater

Real wastewater originating from processes utilized for glucosamine production was used in this study. The wastewater featured with a low pH (0.39 ± 0.05), dark color, fishy smell, and a high Cl^−^ concentration (84.39 ± 1.21 g/L). Other physical properties and chemical composition were not characterized in this study.

Fungi tolerant to salt concentrations equal to or above 100 g/L were adapted gradually into 20, 40, 60 and 80% glucosamine processing wastewater. Peptone, yeast extract and glucose were added into the diluted wastewater to a 20% concentration of the regular YPB (yeast extract peptone dextrose broth) medium. Liquid cultures were performed in 250 mL conical flasks containing 50 mL of growth medium inoculated with 5 × 10^5^ conidia and incubated at 28°C on an orbital shaker at 180 rpm with three individual flasks dedicated for each time point. Conidia collected from low wastewater-containing medium were used for the subsequent culture. Culture supernatants were collected every day from flasks dedicated to each time point by centrifugation at 4,500 g for 30 min. The biomass was weighed after drying and the supernatant was filtered through a 0.22-μm membrane (Millipore, China) at 4°C. The cleared supernatants were then aliquoted in 1.5 mL Eppendorf tubes and stored at −80°C.

### 2.6. Acidity-tolerance assay

Acidity-tolerance assay was performed in liquid culture with 80% wastewater as a medium with different pH values (3.5, 4.0, 4.5, 5.0 and 5.6). The cultivation was performed at 28°C at 180 rpm for 7 days, and the fungal biomass, pH values as well as the Cl^−^ concentration in the wastewater were measured daily.

### 2.7. Measurement of chloride removal from the wastewater

The fungal strains capable of growing in 80% wastewater (Cl^−^ concentration of 67.512 g/L) were further studied and their Cl^−^ removal capacity was detected by ion chromatography at room temperature using a Metrohm Model 761 Compact Ion Chromatograph (Metrohm, Herisau, Switzerland) with suppressor module, equipped with an ICSep AN2™ analytical column (250 mm × 4.6 mm). The injection volume was 20 μL. The eluent used was a 1.8 mM Na_2_CO_3_ + 1.7 mM NaHCO_3_ mixture and the suppressor regenerating solution was 0.1 M H_2_SO_4_. The eluent was freshly prepared and filtered through a 20-μm filter before usage. Data acquisition and processing were performed automatically using the integration software MagIC Net™ software 1.1. Standard solutions (GBW (E)082048, China) of known concentrations of Cl^−^ were analyzed in order to create a calibration curve (Supplementary Figure S2).

### 2.8. Microscopic examination and Cl^−^ tracing

Fungal cells were stained using a chloride sensitive fluorescent probe MQAE (*N*-[ethoxycarbonylmethyl]-6-methoxy-quinolinium bromide, Beyotime, China) and then observed with a fluorescence microscope equipped with an imaging system at an excitation wavelength of 360 nm (D-35578 Wetzlar, Leica, Germany).

To observe the distribution of Cl^−^ in fungal cells, MQAE and Congo Red were used to conduct double-staining for the fungal cells. MQAE was loaded first following the manufacturer’s manual. Briefly, the mycelia were washed three times with Krebs-HEPES buffer (0.02 M, pH 7.4), placed onto glass slides immersed in a drop of MQAE (5 mM), incubated for 30 min at 37°C in the dark, and finally washed again using the buffer. The resulting specimens were then stained using 0.01% Congo Red for 10 min, washed with ddH_2_O to remove the excess stain, and then covered with 4% paraformaldehyde fix solution and a coverslip. A Leica TCS SP5 confocal laser microscope (Leica Microsystems, Germany) equipped with epifluorescence microscopy (Leica DMI 6000B microscope) was used to observe the specimens at excitation of 360 nm (emission at 460 nm) and 497 nm (emission at 614 nm) separately.

### 2.9. *In vivo* infection assays using *Tenebrio molitor* and *Galleria mellonella* models

For these experiments, *A. piperis* was grown on Petri dishes containing yeast peptone dextrose agar (YPD) at 28°C. After a 7-day-culture period, conidia were obtained by washing the plate surface with phosphate-buffered saline (PBS; 10 mM NaH_2_PO_4_, 10 mM Na_2_HPO_4_, 150 mM NaCl, pH 7.2) and filtering them through a 40-μm nylon cell strainer (BD Falcon, Franklin Lakes, NJ, United States) in order to remove the hyphal fragments. The conidial cells were counted in a Neubauer chamber. *Tenebrio molitor* larvae exhibiting clear and uniform color and weighing between 70 and 100 mg were selected for the survival studies (de Souza et al., 2015). *Galleria mellonella* larvae were maintained and fed as previously described until reaching 200–300 mg in weight (Silva et al., 2018). The survival curves (virulence assay) were performed through injection of different fungal inocula (10^2^, 10^3^, 10^4^, 10^5^ and 10^6^ conidia/larva). Larvae (10 per each assayed group) were inoculated with fungal conidia using an insulin syringe (10 μL/larva) and incubated at both 28 and 37°C in Petri dishes containing rearing diet. The inoculation was performed by the injection of fungal suspensions into the *T. molitor* larvae hemocoel in the ventral portion at the second visible sternite above the legs or in the last right proleg of the *G. mellonella* larvae (de Souza et al., 2015). Larvae inoculated with sterile PBS were used as control groups. Larvae were assessed daily, up to 7 days, to check their survival, being scored as dead when they displayed no movement in response to touch. Survival analyses were determined using the log-rank test and the Kaplan–Meier survival curves (GraphPad Prism 6). The experiment was conducted in two independent experimental sets.

## 3. Results

### 3.1. Morphology of the mangrove wetland-derived fungi

**Figure 1:**
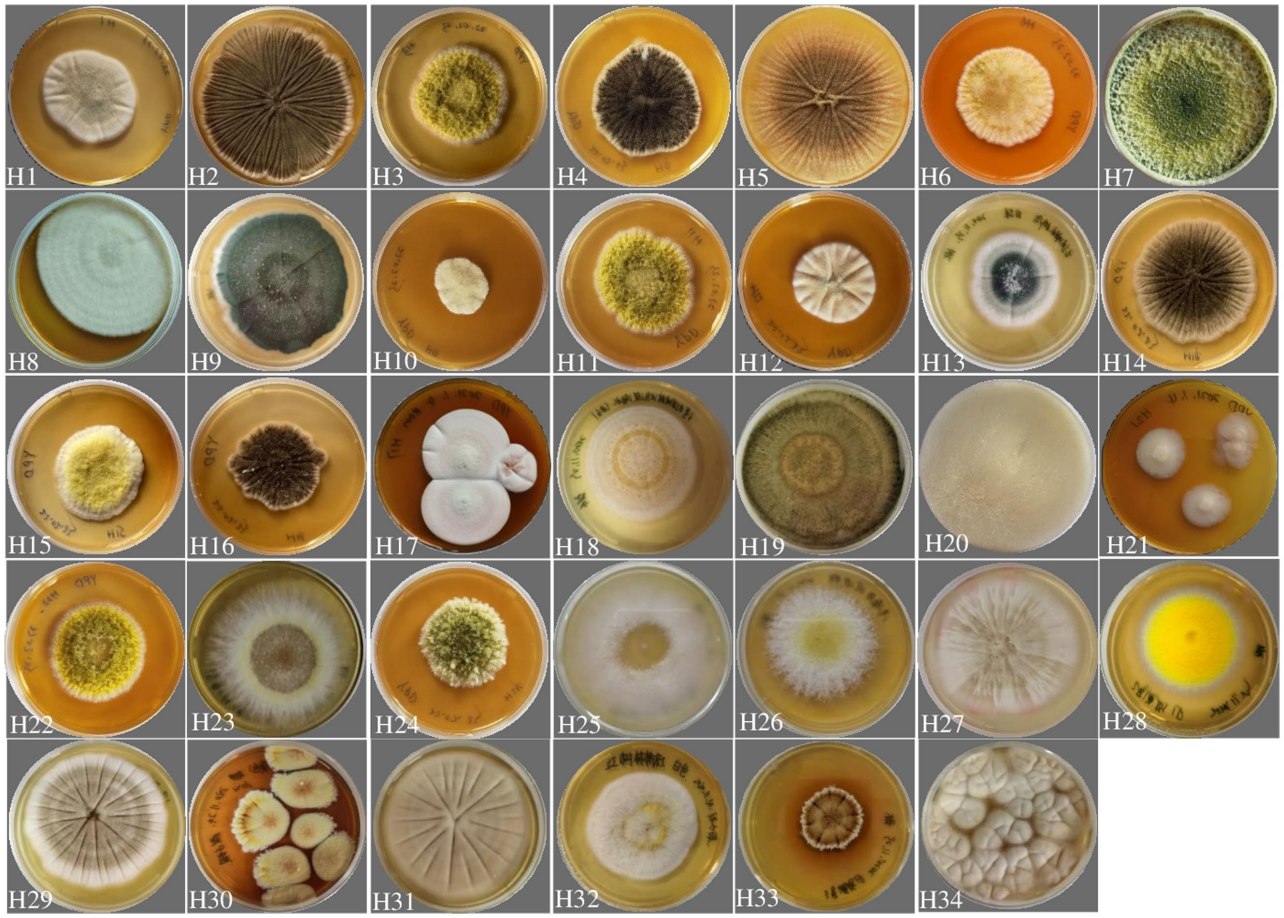
Diversity of fungal colonies isolated from a mangrove wetland located near the industrial park where the high salt wastewater was obtained from. All strains cultivated on YPD medium for 5 days at 28°C.

A total of 34 fungal strains were isolated from the wetlands of a mangrove forest, named H1 to H34 (Figure 1), displayed on a dark background showing the diverse morphology of the fungal population isolated from the mangrove. Detailed taxonomy information for these strains can be found in Supplementary Table S1. The fungal strains’ DNA was successfully amplified using the universal primers ITS1 and ITS4. BLAST searches revealed their identities as members of 3 different phyla (Supplementary Table S1), namely Ascomycota, Basidiomycota and Mucoromycota, in which the Ascomycota accounted for the majority (44.1%). The dominating fungal genera identified in this study were *Aspergillus* (35.3%) and *Penicillium* (20.6%). Representatives of *Trichoderma*, *Furarium, Mucor, Candida, Amanita* and *Talaromyces* genera were additionally identified. Despite *Amanita loosii* is generally recognized as an edible mushroom, the strain identified in this study is not recommended for consumption, considering its origin in an industrial waste environment.

### 3.2. Salt tolerance and chloride removal assay

**Figure 2:**
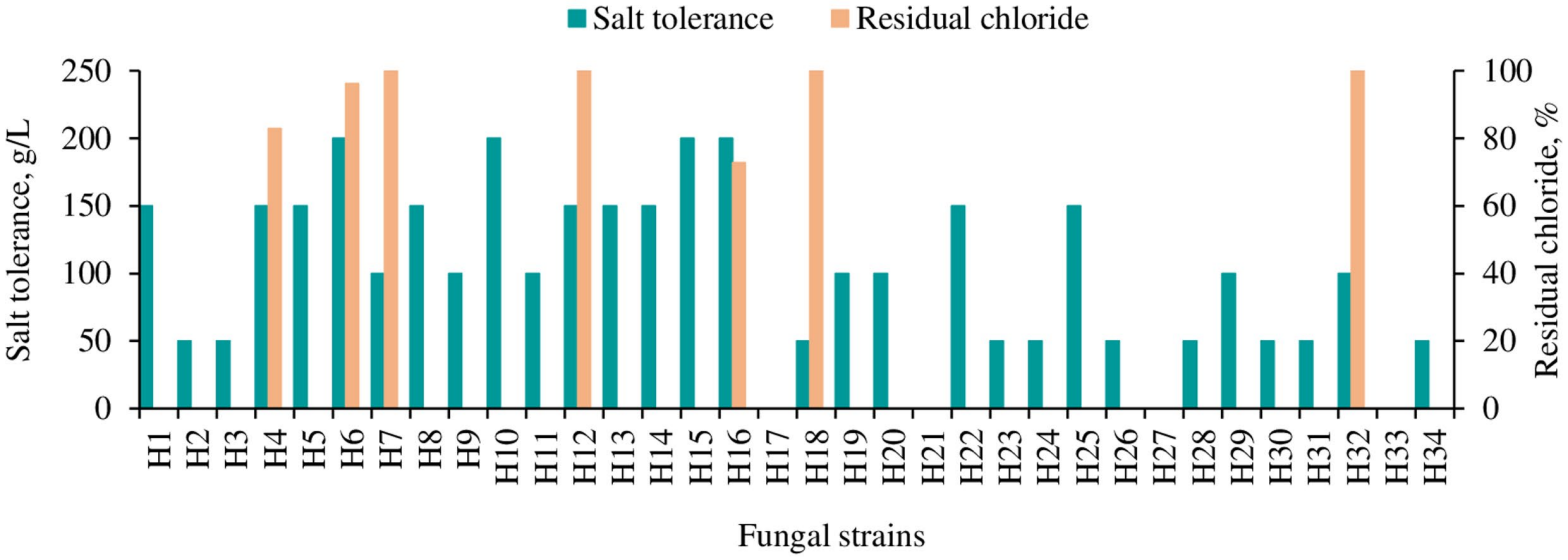
Salt tolerance and chloride removal capacity of different fungal strains isolated from the mangrove forest. Salt tolerance was tested on YPD agar plate. The yellow bar indicates the strains which could grow in 80% wastewater, and residual chloride from the media was measured in liquid culture 2 days post-inoculation with 80% wastewater as a medium (pH 5.6) with Cl^−^ concentration of 67.512 g/L.

An initial screening of these fungal strains for salt tolerance was carried out. The results (Figure 2) showed that 4 strains (H6, H10, H15 and H16) were tolerant to a salinity of 200 g/L, while 8 strains (H1, H4, H8, H12, H13, H14, H22 and H25) tolerant to 150 g/L, 7 strains (H7, H9, H11, H19, H20, H29 and H32) tolerant to 100 g/L, and 10 strains (H2, H3, H8, H23, H24, H26, H28, H30, H31 and H34) tolerant to 50 g/L. Strains H17, H21, H27, and H33 showed tolerance to lower salt levels. However, they were not subjected to further testing because low tolerance was deemed unfeasible for processing shrimp shell effluents, which typically contain salt levels exceeding 50 g/L. The mangrove wetland-derived fungal strains with a salt tolerance level greater than 100 g/L were subsequently gradually adapted into the 80% wastewater and Cl^−^ concentration in the water was measured using ion chromatography. Most of the fungal strains, including those tolerant at 200 g/L NaCl on YPD medium, were not able to grow in the wastewater with pH adjusted to 5.6. Only 7 strains (Figure 2, yellow bars, H4, H6, H7, H12, H16, H18 and H32) adapted to the highest wastewater concentration (80% wastewater). Among these strains, H16 (*Aspergillus piperis*) removed the highest amount of Cl^−^ (approx. 30%) from the wastewater.

### 3.3. pH tolerance and Cl^−^ removal capacity assay

**Figure 3:**
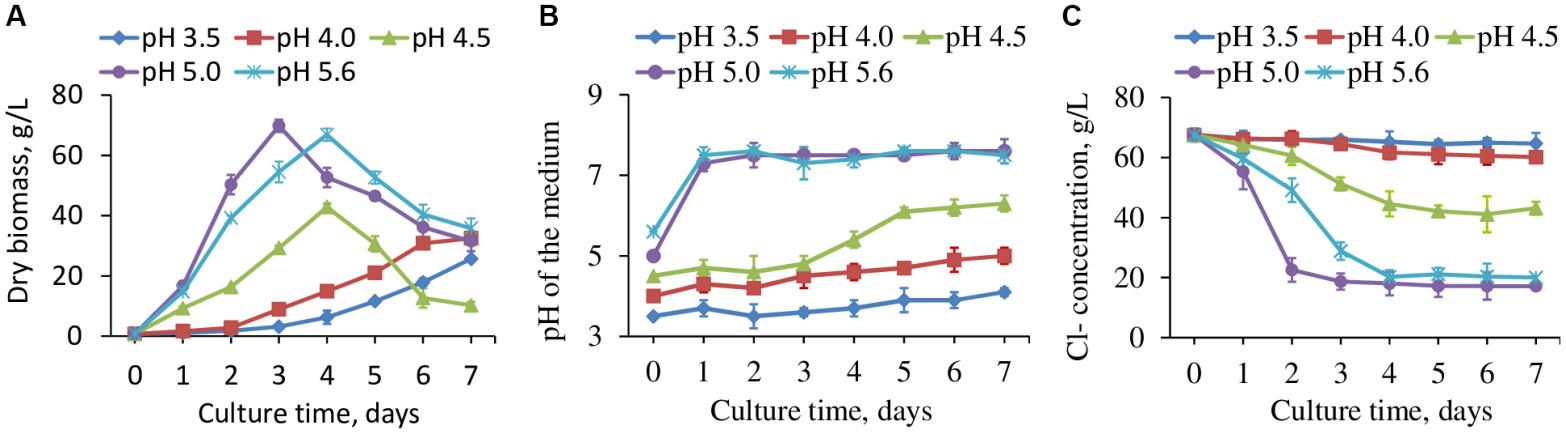
The fungal strain H16 grew in wastewater with different starting pH, the production of biomass **(A)**, pH changes in the water **(B)** and concentration of residual Cl^−^ **(C)**. Data represent mean ± SD (*n* = 3) of three biological replicates. Data for pH 2, 2.5 and 3 were not included as fungi did not grow.

The original pH value of the wastewater was highly acidic (pH 0.39 ± 0.05), in which none of the tested fungal strains survived. A great amount of base (NaOH) was required to neutralize the pH to a level able to support the fungal growth, which would be problematic at an industrial scale. To determine the lowest possible pH for fungal growth, the pH of the wastewater was adjusted to 2.0–5.0, 0.5 intervals and 5.6 to assess viability for strain H16 that had the highest chloride removal. It turned out that H16 could survive down to pH 3.5, with a slow growth rate. Wastewater with pH 5.0 supported the best fungal growth (Figure 3A) and the fungal strain was able to neutralize the acidity of the medium as biomass increased (Figure 3B). Interestingly, while H16 neutralized the acidity of the medium at similar rates at pH 5.0 and pH 5.6, chloride removal was more rapid at pH 5.0 than it was at pH 5.6 (Figure 3C), and the biomass peaked earlier (Figure 3A).

The removal of Cl^−^ was observed to occur in conjunction with fungal exponential phase (Figures 3A,B). When normalized to biomass, chloride reduction varied by days among the tested pH range (Supplementary Figure S1). The fungus apparently tended to reduce more Cl^−^ in a strong acidic environment, as seen from the reduction rates at pH 3.5 and 4.0, which were greater than those at higher pHs. However, the values kept decreasing day by day due to slow growth. pH 4.5, 5.0 and 5.6 brought out smaller Cl^−^ reduction rates at the beginning of the cultivation, but the reductions were enhanced gradually with the highest chloride removal per unit biomass by day 7 at pH 4.5. Exploring the interplays between fungal growth, chloride uptake and medium pH will be an interesting avenue for future research.

**Figure 4:**
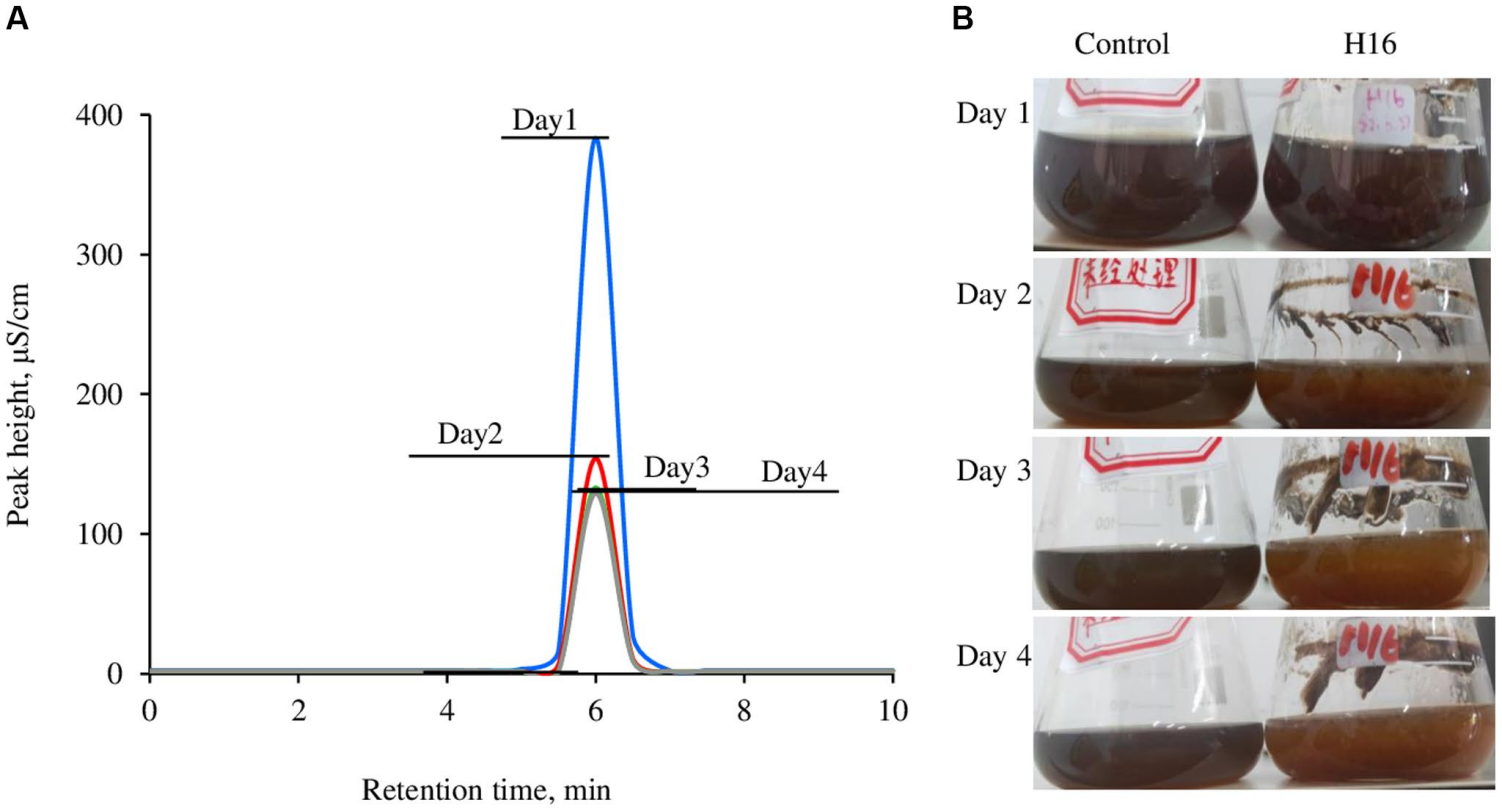
Overlay of Cl^−^ concentration in the wastewater brought by the growth of *Aspergillus piperis*. Cl^−^ was detected using a Compact ion chromatograph **(A)** and the changes in media color over the culturing time **(B)**. All the samples were diluted 500 times before measurement. The wastewater was used as a control. The individual ion chromatographs were available in the Supplementary Figure S3.

The wastewater had a dark color (Figure 4), low pH (1.7 in this study) and a high Cl^−^ concentration (Figure 3C). After adjusting to pH 5.0 using NaOH, strain H16 was able to clarify the broth in pace with the fungal growth, along with the decrease in the Cl^−^ concentration (Figure 4). A visible lightening in the color of the wastewater was also observed, suggesting that other components in the wastewater were remediated by the fungi, but the identification of which was not within the scope of this study. At day 3 post-inoculation, the Cl^−^ concentration in the broth dropped to 17.478 g/L from the original level of 67.512 g/L, corresponding to a 74.11% Cl^−^ removal. Further investigations are needed to lower the Cl^−^ concentration to less than 5 g/L, creating conditions conducive for the growth of most microorganisms. This, in turn, will enable subsequent bio-treatment processes aimed at reducing the chemical oxygen demand (COD) and biochemical oxygen demand (BOD) in the wastewater (Liu et al., 2020).

### 3.4. Distribution of Cl^−^ in fungal cells

**Figure 5:**
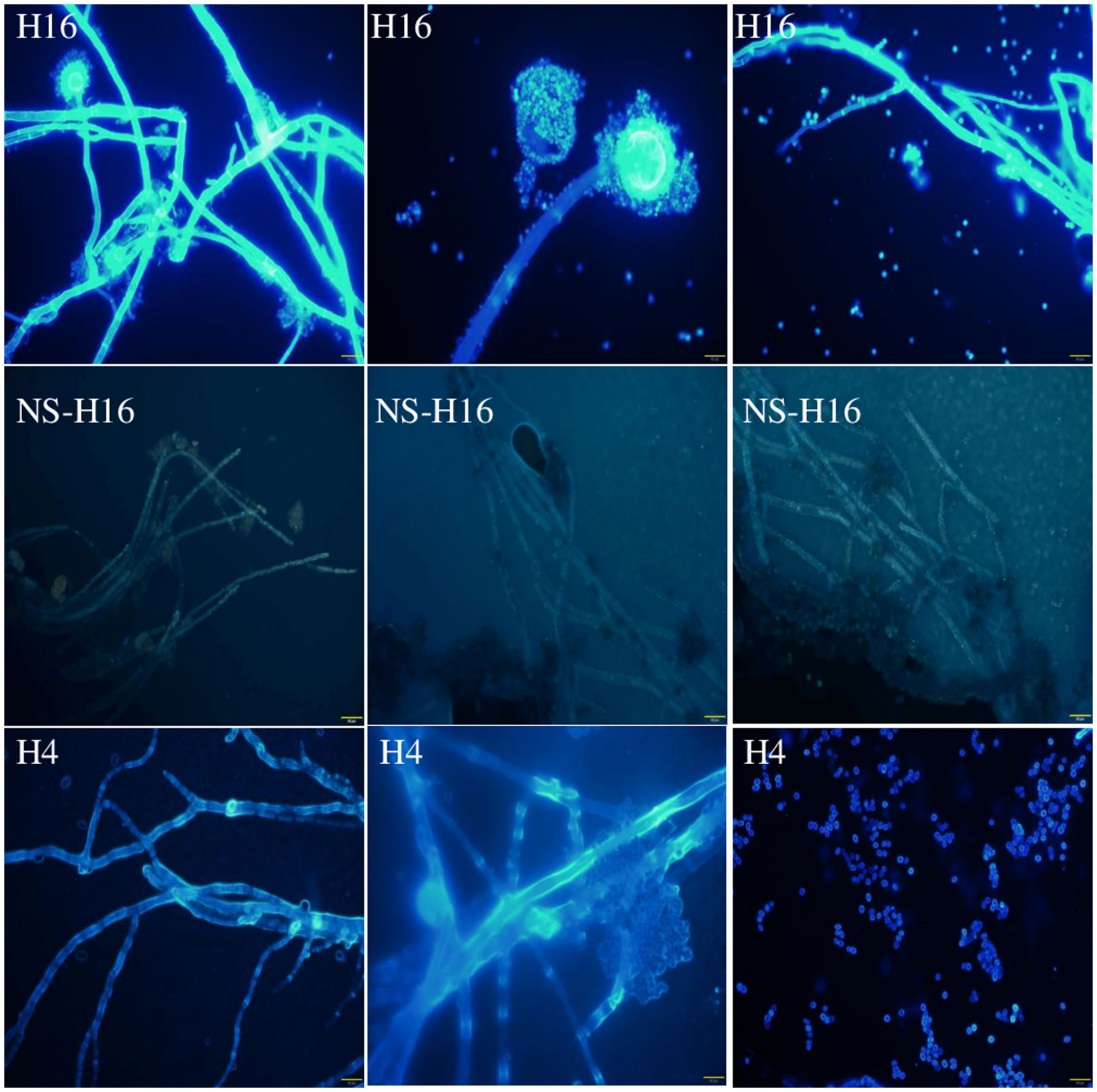
Observation of *A. piperis* stained with Cl^−^ fluorescent probe MQAE (H16). The unstained fungus was used as a control to show the fluorescence of Cl^−^ (NS-H16). H4, growing in wastewater and taking up less Cl^−^ was used as a control as well (H4). The brightness of the NS-H16 images was enhanced 40% to make visible. Magnification: ×400. Scale bars = 20 μm.

**Figure 6:**
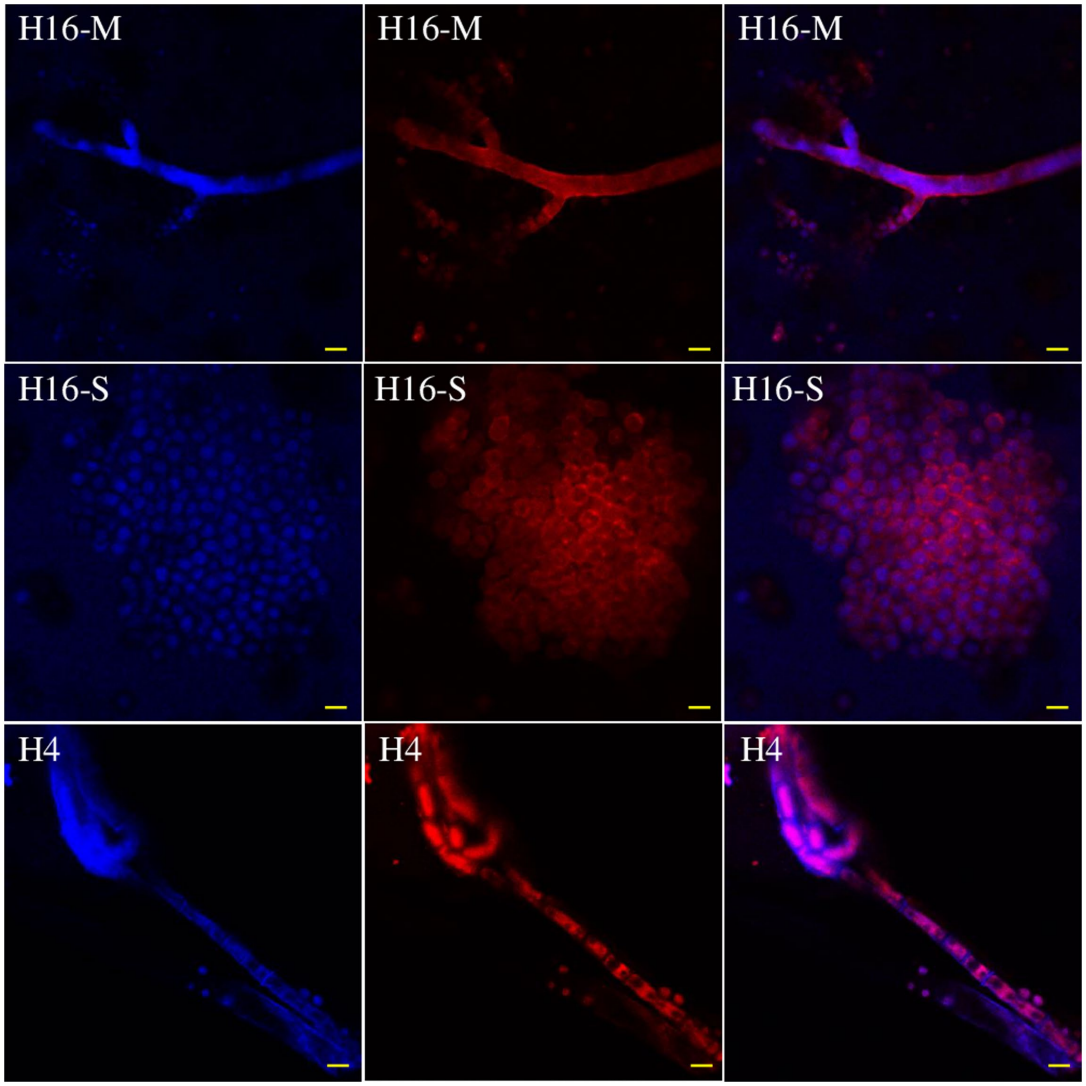
Distribution of Cl^−^ in the mycelia (H16-M) and spores (H16-S) of *A. piperis* 3 days post-inoculation observed using a confocal laser microscope. H4, growing in wastewater and taking up less Cl^−^ was used as a control to verify the staining protocol (H4). Magnification: ×400. Bars = 20 μm.

The Cl^−^ and fungal cells were stained with MQAE (blue) (Figure 5) and/or Congo red (red) (Figure 6) fluorescent stains, and observed using a fluorescent microscope and a confocal laser microscope separately. MQAE is a sensitive chloride ion indicator, and the fluorescence was observed throughout the fungi, including both the hyphae and spores, distributed evenly in the cells, suggesting a strong uptake from the wastewater by strain H16, consistent with the reduction in media Cl^−^ content (Figures 3, 4). Compared to strain H4, which had a reduced chloride clearance ability than H16 (Figure 2), the fluorescence intensity observed was lower (Figure 5). Confocal imaging with Congo Red, which bound to the glucans in the fungal cell wall, showed markedly different outcomes (Figure 6); chloride ions entered H16 cells, while being bound to the exterior of H4 cells. Further studies will be necessary to understand the mechanism behind the uptake of Cl^−^ into the fungal cells.

### 3.5. Virulence capability of *Aspergillus piperis*

**Figure 7:**
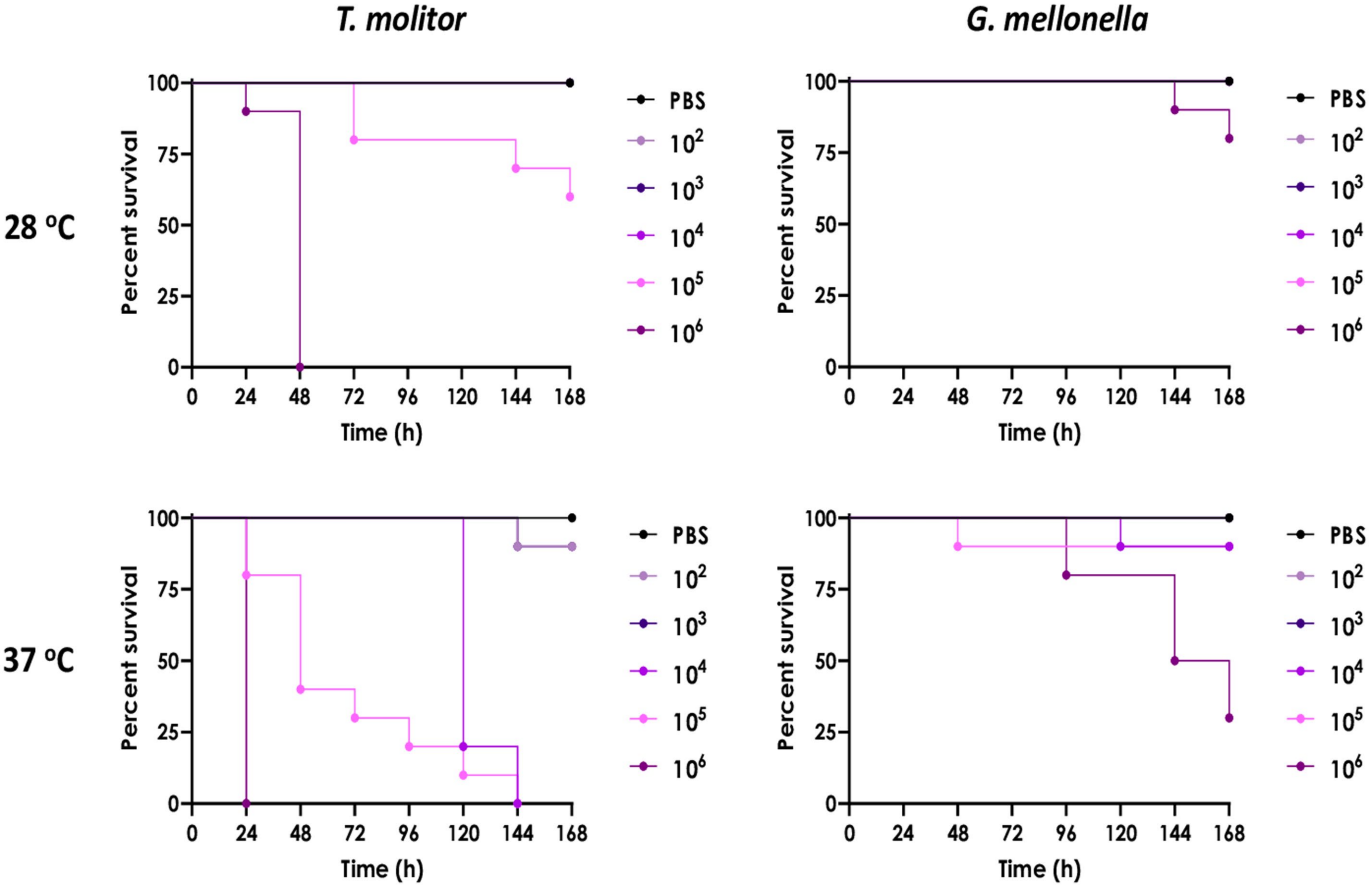
Comparison of *A. piperis* virulence in *Tenebrio molitor* and *Galleria mellonella* larvae infected with different fungal inocula (10^2^, 10^3^, 10^4^, 10^5^ or 10^6^ conidia/larva, *n* = 10 per condition) and incubated at both 28 and 37°C up to 7 days. PBS is used as a control for physical injury caused by inoculation, demonstrating the lack of larval mortality. The results are expressed as percent survival in comparison to uninfected larvae.

The pathogenicity of the isolated *A. piperis* was investigated using *T. molitor* and *G. mellonella* virulence models at both 28 and 37°C. The results of the present assay showed that mortality in both insect models was typically dose-dependent, with doses lower than 1 × 10^5^ conidia having little to no effect on larval mortality within 7 days after fungal inoculation (Figure 7). The fungal infection capability was also found to be modulated by temperature, as incubation at 37°C (host temperature) led to more significant larval killing induced by *A. piperis* in both insect models compared to 28°C (an environmental temperature; Figure 7). It is worth noting that *G. mellonella* larvae were found to be less sensitive to *A. piperis* conidial infection than *T. molitor* larvae under both tested temperatures (Figure 7).

## 4. Discussion

The removal of Cl^−^ from seafood shell processing wastewater using a biological method is highly sought after due to the high global consumption of seafood, such as crabs, lobsters and oysters. By discovering new biological solutions to remediate the high chloride-containing wastewater, recycling efforts for shell wastes can be improved to achieve better sustainability outcomes. In this work, the fungal strain H16, of *A. piperis* genus, isolated from a mangrove swamp near the high-salt wastewater-producing industry, demonstrated the capability to bioremediate the high chloride content of the wastewater. Many fungi have been reported to be halophilic (Musa et al., 2018). Among them, the most salt-tolerant is the basidiomycete fungus *Wallemia ichthyophaga,* which can tolerate as much as 320 g/L of NaCl and grows fast in a high-salt environment (Jančič et al., 2016; Musa et al., 2018). However, there are no reports regarding their capabilities of growing in shell processing wastewater. Furthermore, the wastewater from shell processing not only features a high Cl^−^ concentration but also contains heavy oligosaccharides, COD, BOD, oxidized proteins, and pigments with an extremely dark color and low pH (Gincy et al., 2021). Strain H16 not only demonstrated the ability to survive in the 80% wastewater but also effectively reduced the chloride content and diminished the pigmentation of the wastewater, suggesting multiple modes of bioremediation. Some microorganisms are capable of maintaining different pH levels across the plasma membrane, demonstrating a pH optimum for growth and another pH optimum for tolerance in extreme environment (Baker-Austin and Dopson, 2007). This homeostasis is supported by mechanisms such as organic acid, DNA and protein repair systems, and complex cell wall structures, including reversed transmembrane potentials, highly impermeable cell membranes, and secondary active transport systems (Baker-Austin and Dopson, 2007). Further study showed that the tolerance of acidophilic bacteria to Cl^−^ was highly greater at pH 3.0 than at pH 2.0, although the maximum Cl^−^ resistance level varied greatly between species (Carmen and Barrie, 2018). In our study, *A. piperis* strain H16 demonstrated more efficient Cl^−^ removal from wastewater at pH 5.0 than at pH 3.0 (Figure 3C). Further investigation into nitrogen and carbon conversion will provide insight into the underlying mechanisms. *A. piperis* strain H16 was also able to neutralize the low pH environment of the medium, reducing the amount of base required to transform the highly acidic wastewater environment into a viable one (Figure 3B). The phenomenon has been observed in some fungal species such as *A. nidulans* (Vylkova, 2017), *Saccharomyces cerevisiae* (Palková et al., 2002), and *Candida albicans* (Davis et al., 2000). The increase in pH is believed to be linked to ammonia production, as suggested by existing literature (Vylkova et al., 2011; Vylkova, 2017). Ammonia can be produced either through amino acid catabolism under carbon deprivation conditions or *via* the reduction of nitrate/nitrite under nitrogen catabolite repression mode (Palková et al., 1997; Alkan et al., 2008). Further investigations will be necessary to understand whether strain H16 of *A. piperis* employs a similar mechanism to neutralize the low pH environment.

Chloride ions were sequestered into *A. piperis* (strain H16) rather than being biosorbed, as observed using a confocal laser microscope (Figure 6). Microorganisms exhibit varying responses in salty conditions. Some may succumb to osmotic pressure (Mille et al., 2005), while others some maintain low intracellular salt content relative to the extracellular medium and regulate their metabolism and membrane fluidity accordingly (Plemenitaš et al., 2008). Some microorganisms adsorb and immobilize ions at the cell surface or within biofilms, akin to the phenomenon observed in fluoride ion removal (Yao et al., 2009; Silva et al., 2019). Additionally, some microorganisms sequester and convert the chloride ions into organic chlorine compounds, such as polychlorinated halogenated alkanes, chloroacetic acid and halogenated aromatic compounds (Geng et al., 2009). Further investigation is warranted to elucidate the mechanism by which strain H16 of *A. piperis* adapts to high intracellular chloride concentrations and the specific compounds into which it channels chloride. Previous research has highlighted the involvement of certain enzymes in the biological conversion of Cl^−^ to organic chloride, including S-adenosyl-L-methionine (SAM) chlorinase, SAM methyltransferase, SAM halogenase (Atashgahi et al., 2018), flavin-dependent halogenase (Hopwood, 2012), and chloroperoxidase (Bengtson et al., 2013). A search of the NCBI database for *A. piperis* proteins reveals the presence of four SAM methyltransferase enzymes (GI: 1407039482, GI: 1419162826, GI: 1407048220, and GI: 1407048162). This finding suggests that *A. piperis* might employ SAM methyltransferases to process the excess chloride ions, indicating a potential pathway for chloride ion utilization. However, the overall pathway in fungi remains largely unknown and warrants further studies.

To the best of our knowledge, there are few reports regarding the pathogenesis of *A. piperis*. In this study, larvae of *G. mellonella* and *T. molitor* were employed to investigate this concern. These larvae are highly convenient in *in vivo* models for a variety of research purposes as these invertebrates possess a humoral immune response that is highly compatible with that of mammals. Examples of studies using these insect models include assessing the activity and toxicity of antimicrobial agents, evaluating the virulence capability of microbial agents, and studying the immune response to pathogens (Piatek et al., 2021). In addition, insect larvae are also inexpensive to purchase and house, easy to inoculate, and their use is not subjected to legal or ethical restrictions. Due to these numerous advantages, insect models have become widely adopted in both academic and industrial research settings (Piatek et al., 2021). The fungus isolated in the present study (*A. piperis*) was observed to exhibit low virulent, which was dependent on cell density and temperature (Figure 7). However, it is important to note that every microorganism has the potential to become a pathogen under specific host and environmental conditions. For example, *Saccharomyces cerevisiae*, a yeast species commonly used in the production of bread, pizza and wine, was described as an opportunistic pathogen recently and might cause sepsis in immunosuppressed individuals (Ramos et al., 2023). In contrast, *A. piperis* demonstrates significant potential in bioremediation of pollutants, not only for Cl^−^ as shown in this study but also for heavy metal. Other studies have demonstrated that this fungus is capable of removing heavy metals such as Se (IV), Pb (II), and Zn (II) from water, with a maximum Pb (II) adsorption capacity predicted by isotherm models to be 275.82 mg/g (De Wet et al., 2020; de Wet and Brink, 2021). Further analysis showed that heavy metals were adsorbed on the surface of fungal cells and displaced sodium (Na) and potassium (K) (de Wet and Brink, 2021), which is a different remediation pathway from this study.

Using microorganisms to reduce Cl^−^ concentration in wastewater has been studied in some fungal species. *A. terries* has been found to be able to reduce 43% of Cl^−^ in wastewater samples with a Cl^−^ concentration of 0.478 g/L collected from an urban wastewater treatment plant, while *A. niger* and *Penecillium digitatum* could reduce 22 and 34%, respectively (Kadhim et al., 2021). The authors discovered that the plasma membrane played a role in regulating the elimination of Cl^−^, yet the specific mechanism responsible for this regulation remains unsolved. Sobiecka (2022) used a mixture of both sulphate-reducing and denitrifying bacteria isolated from a petrochemical wastewater sedimentation tank to remove Cl^−^ in a synthetic chloride-rich medium in anaerobic conditions, and achieved a highest removal rate of 15.85% on the third week post-inoculation (Sobiecka, 2022).

## 5. Conclusion

Cl^−^ pollution originates from multiple sources and is considered hazardous at high concentrations. Bioremediation is one of the sustainable approaches to address this issue. Most studies showed that microorganisms conduct Cl^−^ bioremediation through passive processes like adsorption or biosorption. In this study, a mangrove wetland-derived *A. piperis* was found to be able to actively transport chloride ions into their cells, resulting in the removal of 74.11% of Cl^−^ from glucosamine processing wastewater with a salt concentration exceeding 60 g/L in just 3 days. The metabolic processes of Cl^−^ inner the fungal cells deserve further investigation to elucidate the underlying mechanisms and optimize fungal bioremediation techniques. This strain may become an industrially viable chloride bioremediation organism, contributing towards a global push for sustainability.

## Data availability statement

The data presented in the study are deposited in the GenBank repository, accession number OR393858.

## Author contributions

ZH: Data curation, Investigation, Writing – original draft. EM: Methodology, Writing – review & editing. AS: Writing – review & editing, Formal analysis, Supervision. IB: Formal analysis, Investigation, Validation, Writing – original draft. YP: Conceptualization, Methodology, Writing – review & editing. WH: Data curation, Investigation, Writing – original draft. JY: Funding acquisition, Project administration, Supervision, Writing – review & editing.

## Footnotes

^1^ www.ncbi.nlm.nih.gov/BLAST
