# Supplementary material for: Dechlorination of wastewater from shell-based glucosamine processing by mangrove wetland-derived fungi
Source: Front Microbiol. 2023 Oct 13;14:1271286. doi: 10.3389/fmicb.2023.1271286 (PMC10613029; doi:10.3389/fmicb.2023.1271286)
Supplement: Supplementary file 1 [file Data_Sheet_1.pdf]

Supplementary Table 1

No.	Isolate ID (GenBank Accession)	Match identity (%)	Species	GenBank Accession	Phylum, Class, Family
1	H1	99.82	*Penicillium rolfsii*	KM246748.1	Ascomycota, Eurotiomycetes, Aspergillaceae
2	H2	100.00	*Trichoderma asperellum*	MT34117.1	Ascomycota, Sordariomycetes, Hypocreaceae
3	H3	97.00	*Talaromyces stiputatus*	KT240143.1	Ascomycota, Eurotiomycetes, Trichocomaceae
4	H4	100.00	*Aspergillus niger*	MT620753.1	Ascomycota, Eurotiomycetes, Aspergillaceae
5	H5	100.00	*Aspergillus assiutensis*	MT640286.1	Ascomycota, Eurotiomycetes, Aspergillaceae
6	H6	100.00	*Aspergillus flavus*	MT529928.1	Ascomycota, Eurotiomycetes, Aspergillaceae
7	H7	99.81	*Aspergillus japonicus*	EU645662.1	Ascomycota, Eurotiomycetes, Aspergillaceae
8	H8	100.00	*Fusarium solani*	MT447548.1	Ascomycota, Sordariomycetes, Nectriaceae
9	H9	100.00	*Penicillium oxalicum*	MT588795.1	Ascomycota, Sordariomycetes, Nectriaceae
10	H10	100.00	*Aspergillus terreus*	MT530257.1	Ascomycota, Eurotiomycetes, Aspergillaceae
11	H11	100.00	*Fusarium falciforme*	MN907498.1	Ascomycota, Sordariomycetes, Nectriaceae
12	H12	98.22	*Penicillium pulvillorum*	MH865335.1	Ascomycota, Sordariomycetes, Nectriaceae
13	H13	98.74	*Penicillium oxalicum*	MT033032.1	Ascomycota, Eurotiomycetes, Aspergillaceae
14	H14	100.00	*Aspergillus costaricensis*	MT558927.1	Ascomycota, Eurotiomycetes,

					Aspergillaceae
15	H15	100.00	*Talaromyces stipitatus*	MH443359.1	Ascomycota, Eurotiomycetes, Trichocomaceae
16	H16	100.00	*Aspergillus piperis*	MT588789.1	Ascomycota, Eurotiomycetes, Aspergillaceae
17	H17	100.00	*Penicillium verruculosum*	JQ717338.1	Ascomycota, Eurotiomycetes, Aspergillaceae
18	H18	94.65	*Amanita loosii*	JQ512094.1	Basidiomycota Agaricomycetes Amanitaceae
19	H19	100.00	*Trichoderma harzianum*	MK910066.1	Ascomycota, Sordariomycetes, Hypocreaceae
20	H20	100.00	*Mucor indicus*	MK967563.1	Mucoromycota, Mucoromycetes, Mucoraceae
21	H21	100.00	*Candida tropicalis*	CP048737.1	Ascomycota, Saccharomycetes, Debaryomycetaceae
22	H22	100.00	*Aspergillus tubingensis*	MT645322.1	Ascomycota, Eurotiomycetes, Aspergillaceae
23	H23	100.00	*Talaromyces stiputatus*	MH443359.1	Ascomycota, Eurotiomycetes, Trichocomaceae
24	H24	99.81	*Aspergillus oryzae*	MN856407.1	Ascomycota, Eurotiomycetes, Aspergillaceae
25	H25	97.02	*Aspergillus carbonarius*	LC573577.1	Ascomycota, Eurotiomycetes, Aspergillaceae
26	H26	98.83	*Trichoderma afroharzianum*	MN518415.1	Ascomycota, Sordariomycetes, Hypocreaceae
27	H27	92.81	*Aspergillus oryzae*	MN856407.1	Ascomycota, Eurotiomycetes, Aspergillaceae
28	H28	95.41	*Aspergillus lanosus*	MH865974.1	Ascomycota, Eurotiomycetes, Aspergillaceae
29	H29	100.00	*Trichoderma*	KY069811.1	Ascomycota,

			atroviride		Sordariomycetes, Hypocreaceae
30	H30	99.64	*Penicillium tanzanicum*	OP358460.1	Ascomycota, Eurotiomycetes, Aspergillaceae
31	H31	98.21	*Penicillium pulvillorum*	MH865967.1	Ascomycota, Eurotiomycetes, Aspergillaceae
32	H32	97.53	*Trichoderma koningii*	KT192056.1	Ascomycota, Sordariomycetes, Hypocreaceae
33	H33	100.00	*Aspergillus terreus*	NR_131276.1	Ascomycota, Eurotiomycetes, Aspergillaceae
34	H34	100.00	*Penicillium* sp. Isolate 26	MT588790.1	Ascomycota, Sordariomycetes, Nectriaceae

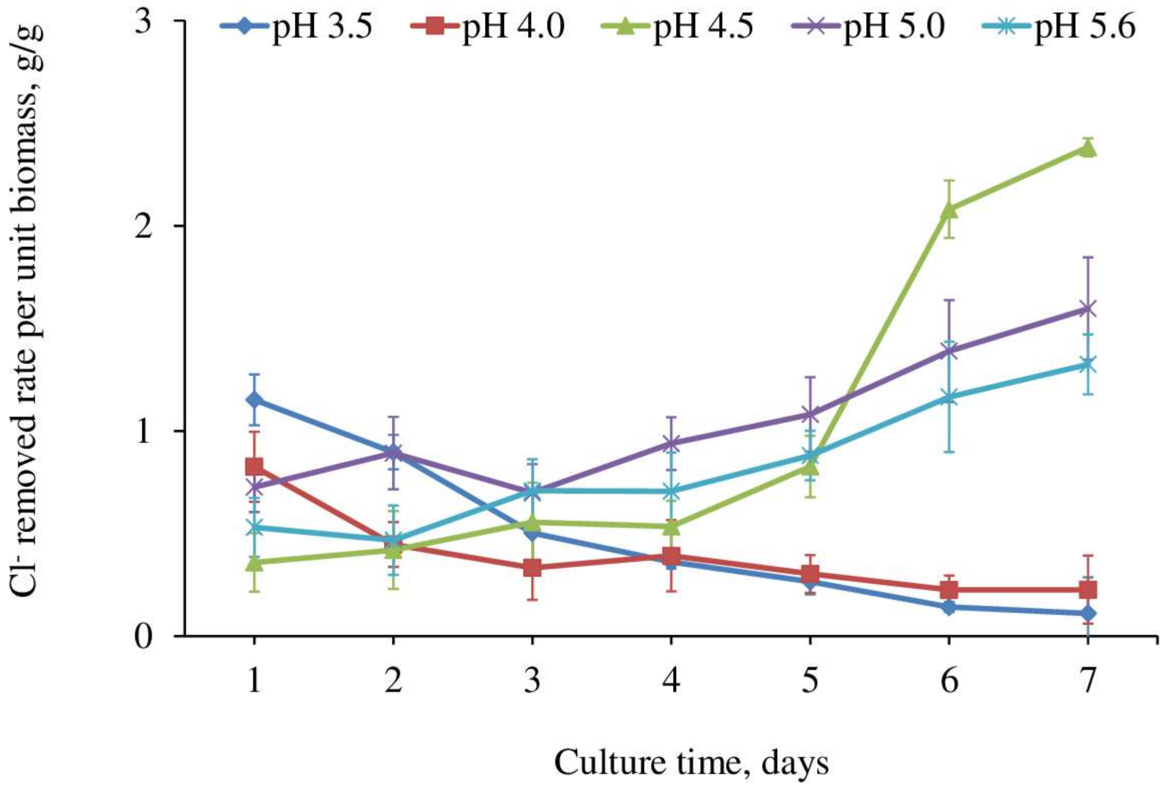

Supplementary Figure 1

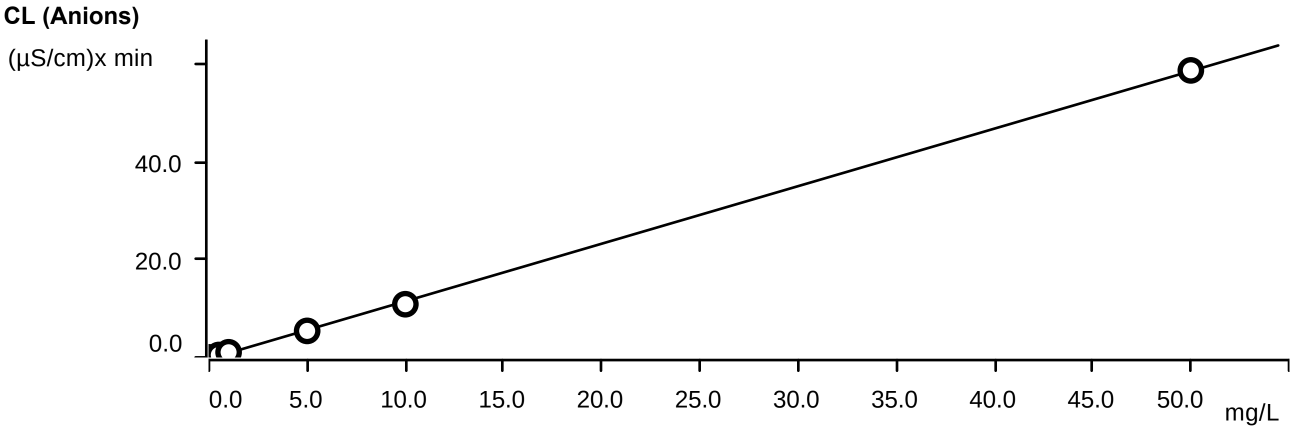

Supplementary Figure 2

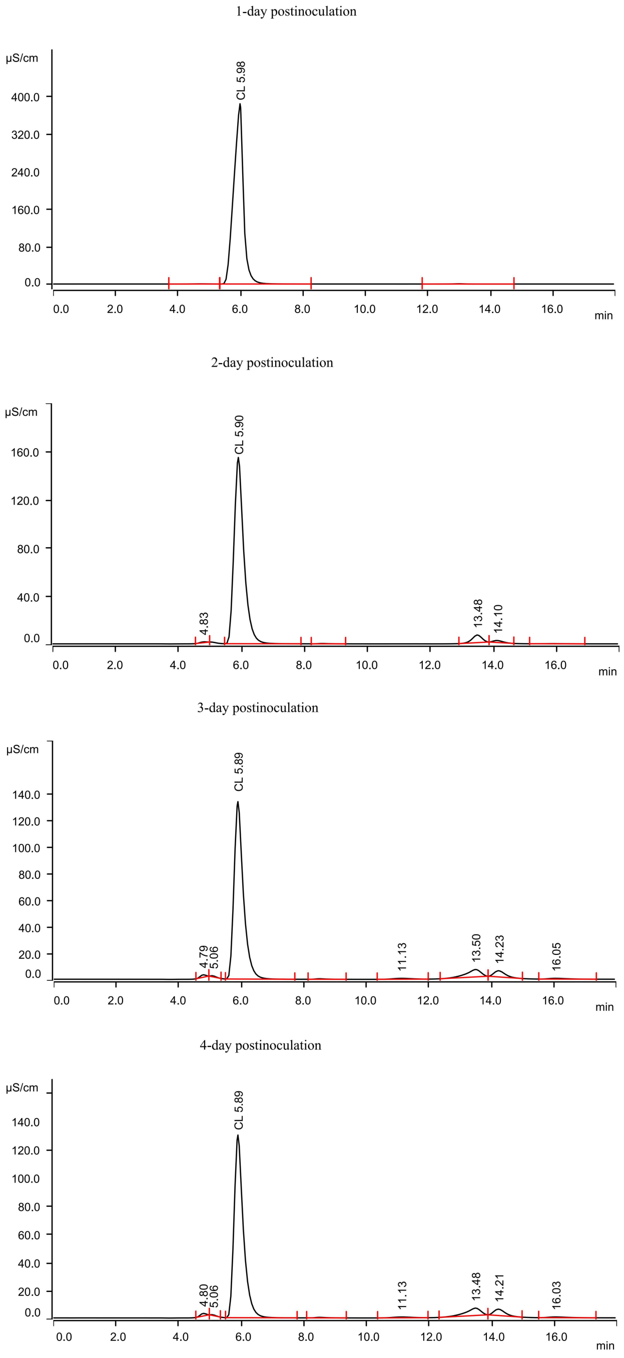

Supplementary Figure 3
